# The Ties That Bind: Materiality, Identity, and the Life Course in the “Things” Families Keep

**DOI:** 10.1177/0363199017746451

**Published:** 2018-03-15

**Authors:** Liz Gloyn, Vicky Crewe, Laura King, Anna Woodham

**Affiliations:** 1 Royal Holloway, University of London, Egham, Surrey, UK; 2 University of Sheffield, Sheffield, UK; 3 University of Leeds, Leeds, UK; 4 King’s College London, London, UK

**Keywords:** family archive, identity formation, archives, material culture, family, history, gender, people–object interactions, socialization

## Abstract

Using an interdisciplinary research methodology across three archaeological and historical case studies, this article explores “family archives.” Four themes illustrate how objects held in family archives, curation practices, and intergenerational narratives reinforce a family’s sense of itself: people–object interactions, gender, socialization and identity formation, and the “life course.” These themes provide a framework for professional archivists to assist communities and individuals working with their own family archives. We argue that the family archive, broadly defined, encourages a more egalitarian approach to history. We suggest a multiperiod analysis draws attention to historical forms of knowledge and meaning-making practices over time.

As Orhan Pamuk argues in his “Modest Manifesto for Museums,” “the future of museums is in our own homes.”^1^ He suggests that a more democratic, egalitarian and just approach to museum making would focus on small museums set in neighborhoods and homes rather than large, state-led institutions. Such an approach would enable individuals’ stories to be told in place of a focus on the histories of nations. Indeed, families and individuals have long collected and, perhaps, even curated objects and stories relating to their own lives. Further, objects held in family archives play an important role within the wider landscape of memory and history. This article uses an interdisciplinary approach to consider how living generations use physical artifacts to interact with long-dead and not-yet-born family members and to curate strong, cross-temporal family identities. As such, it builds on understandings of family identities over time and on archiving and curating practices in three different societies. We argue that considering the way families preserve, keep, and pass on items, along with their associated stories, in its own terms is crucial. Rather than seeing family archiving solely through the prism of institutional archiving practices and therefore understanding it as a poor cousin to the formality and rigor of a formal archive, we suggest that family archives are in part valuable precisely because of their fluid, chaotic, and informal nature. The long-term historical perspective this article adopts allows us to see what, why, and how families have kept objects and how these actions change in different cultural contexts.

This research forms part of the Arts and Humanities Research Council funded Family Archive Project, which explores the past, present, and future of the notion of the family archive and considers what families need to curate their own archives. The examination of archival and curatorial practices that take place outside formal cultural and heritage organizations, both historically and currently, helps historians and academics concerned with archives and heritage understand the dynamics at work in such activity as well as helping professional archivists and curators respond to the needs of the so-called citizen archivists or citizen curators.^2^ This article offers a historical perspective on the family archive, exploring how individuals and family units collect items representing their past and preserve them for their future, and presents a range of methodological approaches to studying the material culture of family life.

After considering the methodology used to combine insights from a range of academic disciplines and addressing the issues around defining an archive and a family, this article considers four main ways in which families have approached collating and curating their possessions. These themes consider the way people interact with objects, the significance of gender within the family archive, how family archives shape the formation of identity, and the role the archive plays in marking the progression and form of the social life course. These central concepts help illuminate the function of the family archive; in concluding, we offer some thoughts on the future implications of this work for researchers and curators.

We argue that traditional historical approaches to understanding and valuing archival practices privilege state-driven modes of history-making, formal and institutionalized ways of thinking, and masculine and patriarchal forms of knowledge. Historically, the formal record of the national or regional archive, and the apparently rational mode of knowledge it represents, has been viewed as superior to the private archives of “ordinary” people. However, we argue that by redefining what counts as an “archive,” historians are able to value and recognize the more messy types of knowledge held within social groupings such as families rather than relying solely on the “objective” knowledge of the professional institution; this change of perspective allows new avenues for learning about the past to emerge. The oral history traditions of non-Western societies offer one potential alternative model; their historical practice preserves not only the facts, figures, and key events of their pasts but emotions, feelings, social dynamics, and the more subjective aspects of individual lives.^3^ Yet such processes are at work within Western families and other social groups, even if they are less sophisticated and formalized. As well as taking nonelite groups as subjects of history seriously and locating the voices of those individuals where possible, we argue for an “objects from below” and “archives from below” approach, which engages with material culture and the archival practices of individuals and families. What our deep chronological perspective demonstrates is that there are long historical roots of different ways of valuing knowledge, existing outside state structures, and other formal institutions.

## An Interdisciplinary Methodology

In recent years, a massive growth of interest in family history and genealogy as a hobby, seen in the popularity of television programs like *Who Do You Think You Are?* and websites such as ancestry.com, reflects a public fascination with personal and family pasts. This form of “popular” history has its roots in the practice of families using stories and memories to contribute to a particular sense of shared familial identity. By reconsidering how these narratives are curated and circulated, we argue that a deep historical approach reveals the importance of exploring processes of archive formation which take place away from civic narratives and which challenge the traditional privileging of words over objects.

The methodology for this article sees the straightforward existence of families and objects as constants across time but highlights that their nature and social construction change very significantly depending on chronological period and social context. We explore whether the family archive was a concept that can be applied across time and whether interlinking themes emerge about how humans relate to things in this particular situation. Such a cross-period approach has been successfully used to bring out commonalities in, for example, the study of the ancient library.^4^ Applying that lens here allows us to trace continuities in the fluidity of domestic practices without limiting them to the assumptions associated with a single period.

We use three case studies to explore different models for the family archive from a historical perspective. The first case study examines ancient Roman approaches to preserving family histories in the early imperial period, drawing out culturally specific ways of creating a shared inherited narrative.^5^ The second case study reviews archaeological evidence for the reuse, curation, and repair of domestic artifacts (including jewelry, ceramics, and other personal items) between the fifth and fifteenth centuries AD in Britain to reveal changing practices of curation and repair. The third case study uses a selection of autobiographies relating to life in the first half of the twentieth century in Britain to find evidence of how working-class people chose to preserve and document their family histories including what items were kept within the family home and by whom. Each case study brings its own challenges of negotiating the conventions surrounding the language of archives in each discipline; these issues helped highlight patterns in the other case studies which would otherwise have been missed.

Within classics and papyrology, surviving archives are often associated with the machinery of government in some way;^6^ while it is obvious that families *had* archives containing at the very least important legal documents, few of these survive, and little is said about them in the surviving literary sources. This difficulty is compounded by the comparatively late arrival of the public library during the late Roman Republic. The need for individuals to collect private research libraries, which were often quite considerable, makes the preservation of private documents among the elite vanish under a more traceable concern with acquiring copies of rare books.^7^ However, the practice of preserving ancestral masks provides an unexpected door into the memorialization of previous generations through objects and text, but one that the current literature does not consider in terms of family archives.^8^

For our second historical case study, which focuses on family heirlooms in the early Anglo-Saxon and medieval periods in Britain, the term archive is rarely applied, certainly in domestic contexts, although it can be used to describe ecclesiastical or royal collections. We can, however, consider the role of objects as familial items or heirlooms, which may have had biographical associations and been passed down through a number of generations. Archaeologists of this period have recently begun to explore the concept of “heirlooms” in Anglo-Saxon and medieval households; this work allows us to examine the processes of “curation” or “archiving” of artifacts by families.^9^ Items that qualify for the status of heirloom may include both ancient objects, such as centuries-old Roman jewelry and household items deposited with the dead, and household and personal items found on settlement sites that show evidence of curation and repair over several generations. There are two key methods for identifying potential heirlooms archaeologically: firstly, where objects are considerably older in stylistic terms than the securely dated deposit in which they are found and, secondly, where the wear, breakage, repair, and alteration of artifacts indicate their long-term circulation and use over more than one generation.^10^ This article includes both types of object in its concept of “family archiving.” In taking a comparative approach, we are also aware that heirlooms made from perishable materials such as wooden furniture or textiles are unlikely to have survived archaeologically.

For working-class families in twentieth-century Britain, objects were one means through which they could create a particular family identity. Our third case study examines published autobiographies to understand the role of objects as compared to other methods for preserving family history. Autobiographies are particularly useful in this kind of research for two reasons: they provide information about family archiving practices and they are a medium through which to directly preserve family histories and “archives.” Autobiographers were consciously “curating” and “archiving” their own personal pasts and often that of their families too. The dedication of Ted Walker’s autobiography, for example, reads “For My Family—Present, Past and Future.”^11^ Using these documents can tell us both about which objects individuals kept, treasured, and used to remember and their reflections about this matter in older age as they write, directly and indirectly engaging with the very processes of making history. Autobiographers often use their writing to document particularly significant histories, to tell these stories while they can, or to set the record straight in later life. As such, these texts are useful for thinking about subjectivity and family archives; though published for a particular audience, they are also highly revealing in terms of the ways in which individuals make sense of their own life and want to present that history to others.

## How Do We Define a Family Archive?

As part of our acknowledgment of the importance of informality and chaos to this process, we take a broad view of how to define a family archive and, indeed, a “family.” While we focus on tangible items that families might curate and pass down, they cannot be artificially separated from intangible items; often unremarkable objects become valuable precisely because of the role they play in a remembered family anecdote, event, or connection to an individual. As Jeremy Seabrook notes of family photographs, they “serve to reinforce the oral tradition, give it fresh impetus and validation.”^12^ This is expressed by Weiner’s anthropological concept of “inalienable wealth,” objects “imbued with intrinsic and ineffable identities of their owners which are not easy to give away.”^13^ Within the field of physical artifacts, the range of items that can go into a family archive is also extremely varied. Some categories of item appear regularly in our different case studies, while others appear specific to a particular time and place. Some objects are very deliberately gathered and saved, while others enter family archives more haphazardly and indeed even accidentally.

Archives are associated in the popular mind with documents, which unsurprisingly appear in many case study examples. The terminology of whether to call collections of documents belonging to private individuals an archive or a dossier has generated debate among papyrologists, but the barriers between the public and the private in such collections that survive from the ancient world are often extremely permeable.^14^ Many archives recovered from Roman Egypt contain a collection of documents used in legal proceedings; a good example is the archive of Gaius Julius Agrippinus, which records a battle over ownership of some mortgaged fields through a range of documents including petitions, legal records, and private letters.^15^

More personal documents also appear in our case studies. In our twentieth-century case study, autobiographers referred to deliberately preserving personal letters from their families. Allan Jobson’s account demonstrates how personal papers could be deeply treasured, in this instance in a very private way, for their connection with deceased family members. He described how his father kept his grandmother’s letters: “A few of grandmother’s letters were preserved by Father. These contain little human touches showing how she hoped he would be able to get home and how pleased the girls would be (his two step-sisters).” He added that “Father kept those letters locked in his box beside his bench and they were not seen by prying eyes until after his death.”^16^

Another common category of object is that of likenesses. While photography was available during the period of only one of our case studies, other periods found ways to represent the human face and remember significant individuals. Elite Romans had a cabinet in their household *atrium* or central hall filled with *imagines maiorum*—masks of illustrious ancestors, probably made of wax.^17^ The *imagines* were complemented by *tituli*, which were inscriptions painted on the atrium wall, possibly with a family tree; these outlined the achievements of each commemorated family member.^18^

Many twentieth-century autobiographers recalled photographs as a key way to recall family histories and preserve them for future generations. Indeed, many of these autobiographies include examples of treasured family photos reprinted for the reader’s benefit.^19^ Including photos within an autobiographical text serves a purpose beyond simple illustration; they have a special place within the production of memory.^20^ However, one might argue that this use of family photography can help create an idealized vision of family life.^21^ Jo Spence highlights this function of family albums and photography, noting the role of the photographer is to “paper over the cracks of the dissent that you see.”^22^ Creating likenesses of a family could therefore be part of a process of idealizing family life, whether that family life was happy or otherwise. For example, one more positive part of Jo Stafford’s often unhappy 1950s childhood was the recollections of evenings when her mother told her children stories of the past:

After we had eaten and settled down around mom’s feet we could sometimes persuade her to get out the photos. Out of the cupboard beside the fireplace came a biscuit-tin containing the beloved snap-shots. Sitting in her chair at the fireside, tin on lap, off she went, chanting the litany we never tired of hearing. Speaking in her soft Black Country accent I hear her still, and see her fingers rummaging through the box as she carefully selected photographs and imparted their history.^23^

Photographs could be also intimidating. Jo recalled staying at her grandmother’s house and described the spare bedroom: “worst of all were the pictures hanging from the picture rail. […] The largest photograph was of my mother’s soldier brother, recently deceased. He was glassy-eyed and stiff and I desperately wanted to escape from his fearful presence.”^24^

Myriad personal and domestic objects, some apparently mundane in nature, could be signifiers of familial identity and memory. In the Anglo-Saxon and medieval periods, repaired and reused personal objects may have served as a way to mediate family relationships. Roman items, including coins, jewelry, and spoons, were deliberately deposited in Anglo-Saxon graves and settlements dating from the fifth to seventh centuries and have also been identified in later medieval burials.^25^ Some of these items may have been found on abandoned Roman sites, but it is also possible that they were passed down through generations that spanned the transition from “Roman” to “Anglo-Saxon.” The antiquity of these objects may have imbued them with perceived amuletic or magical powers, but they may also have been heirlooms, passed from one generation to another. Indeed, the two meanings may have overlapped; if a family member discovered an antique object with perceived protective or magical properties, this could have been treasured and bequeathed to other family members, keeping its origins in the ancient past but gaining familial symbolism in the recent past.

In addition to antique objects, repaired and modified items such as brooches, buckles, and pottery vessels have been noted at numerous fifth- to fifteenth-century cemetery and settlement sites.^26^ These objects may have been curated rather than discarded when broken because they held particular significance relating to an individual’s or family’s identity which it was important to maintain over generations. Pottery vessels offer a helpful illustration of this trend. Roberta Gilchrist draws attention to several examples of decorative ceramic vessels discovered in household deposits in medieval England including a latrine deposit from Winchester dating to the late fourteenth or early fifteenth century, which contained a tankard and two jugs of Saintonge ware, a distinctive decorated style of ceramic from France with a production date of c.1280 to 1330.^27^ The three vessels in this deposit would therefore have been retained for two or three generations (based on a life expectancy of fifty years and a generational length of thirty years). The highly decorative nature of these items may indicate that they were used in family rituals involving drinking and display, perhaps surrounding births, marriages, and deaths. Heirlooms need not have been archaeologically recovered; *Books of Hours*, illustrated personal items used for private devotional study and particularly popular in the fifteenth century, survive in library collections, and often have been expanded with extra pages and inscriptions of family births, marriages, and deaths before being handed down the generations.^28^

In some instances, it was the material, rather than the object itself, that had familial value. In her tenth-century will, a woman named Wynflaed specified that she was leaving an engraved ring and a cloak fastener to her daughter and her “*goldfagan* cup” to her grandson, so that he could enlarge his *beah* or arm ring.^29^ Similarly, recent analysis of metal alloy composites used in the manufacture of saucer brooches in fifth- to seventh-century England suggests that Roman brass artifacts were melted down to add to the alloy.^30^ Saucer brooches, worn by women on the left and right shoulders, were made in matching pairs. It appears that for each pair, an older brass object was divided, with half melted into the composite for each brooch, “so that the ancestral values were passed through the metal into the new saucer brooches.”^31^ The significance of the original brass object was thus transferred into both parts of the new pair. As Katina Lillios has noted in her anthropological research, even fragments of ancestral possessions can hold as much meaning and power as complete artifacts; some parts may be kept by family members and others passed on more widely or deposited with the owner when they die, but each fragment can still represent a person’s or family’s identity or an important event.^32^

The twentieth-century autobiographers mentioned a wide range of family objects including cameo brooches, a grandfather’s sporting medal, and pieces of art made by a family member.^33^ A middle-class writer, Molly Hughes, spoke about the furniture that she and her husband had acquired over the years including “a Welsh dresser from an inn at Aberdovey, and a ‘cubbard dyddarn’ (a large double cupboard) that we found in a cottage hardly bigger than the cupboard itself”; when Hughes built a new house during the interwar period, she asked the builder to create spaces specially for these items, thus shaping her living space around her physical memories.^34^ David Barr even spoke about a piece of plum cake his mother had given him when he left home: “without intending it at the time, I have carefully preserved it all through my life, for sixty-two years, and it is now regarded as a precious relic by my family.”^35^

All these items share the function of maintaining family identity both in terms of continued joint ownership of objects and developing “the construction and transmission of joint memory structures.”^36^ Four themes emerged from the case studies that illustrate the ways in which the objects held in family archives, curation practices, and associated intergenerational narratives reinforce a family’s sense of itself.

## Theme 1: People–Object Interactions

Our first major theme concerns the way in which people interact with objects and applies the “object biography” approach often used in anthropology and archaeology to items held in a family archive. This biographical approach recognizes that objects may have very different meanings and roles over time; like human beings, they experience different stages in their lives including manufacture, trade, being worn, or being put on display.^37^ The example of old family photos and used birthday cards and postcards shows how this approach helps us to understand how meaning is constructed and associated with particular items at different stages in their histories. While cards and photos have a particular meaning when held within a family home, when gathered en masse in a curio or junk shop they lose that significance. However, if one card is purchased and given to a new recipient or added to a collection, then its meaning is refreshed and it joins another family archive with different associations.

These “cycles of significance” associated with objects reflect the ebb and flow of meaning depending on where the object is located and who owns it at any given time. While being possessed by one particular owner gives an item a specific value at a specific moment, the object itself can have many such stages over its existence. The process of assigning meaning to objects is thus “not prior to or independent of social practices but codependent.”^38^ Indeed, the physical traces of use that an object develops during a particular biographical phase may imbue it with even more meaning in the next phase: the patina of repeated rubbing on furniture or jewelry, the folds in a photograph or letter, or breaks and repairs in a vase are proof that an item was handled by earlier generations, offering a tangible link to the past life of the object and past lives of family members.^39^ As Pamuk notes, “the power of things inheres in the memories they gather up inside them, and also in the vicissitudes of our imagination, and our memory.”^40^ Considering items from the family archive in this way acknowledges the varied associations and values that may be placed on objects through the various iterations of an archive.

The archaeological record offers one place to observe this process in action. As discussed above, Anglo-Saxon graves often contained Roman coins, many of which had been pierced to allow them to be worn as necklaces. The coins began their existence as functional tokens of currency but had eventually become items of jewelry to be worn on the body.^41^ Anglo-Saxon communities may have believed that these objects had protective or magical powers, and that apotropaic function gives the coins an additional layer of meaning and significance.^42^ The provenance of these items before they entered the possession of the dead is unclear; they may have been acquired from Romans originally and passed down through generations or retrieved from abandoned Roman sites during the Anglo-Saxon period because of their antiquity or “otherness.” There have been some attempts to distinguish between the two object types, marking the former as heirlooms and the latter as “ancestor objects” (i.e., items of unknown provenance nonetheless valued for being old).^43^ The object biography approach, however, allows us to consider them as *both* heirlooms and ancestor objects; a Roman coin discovered centuries after it was originally lost might have been valued and retained due to its age but could also have been incorporated into a family’s collection of treasured items and even into family stories to be passed down the generations to living family members or buried with the deceased.

The question of pedigree and where an object came from often became attached to books in the Roman period due to the crossover between family archives and personal libraries. One excellent example is the library of the Republican general Lucullus, which he collected on campaign in northern Asia Minor. His son inherited the library upon his father’s death, and the collection remained in the family’s house at Tusculum. In *On the Ends of Good and Evil*, Cicero invents a conversation between himself and Cato set in the library itself, where he hopes that the younger Lucullus will absorb the good lessons represented by the physical artifacts around him (*De Finibus* 3.7-9). The books Lucullus senior collected move from being battle spoils to symbols of the intellectual lineage and moral worth of the next generation of Roman citizens.

The twentieth-century autobiographies also contained many examples of objects with changing or overlapping meanings. Ted Walker actually used the term “family archive” for his family’s cupboard door:

The food cupboard door was a family archive. Every so often I would be made to stand in stocking feet against the door, and I would feel the cold blade of the fish-slice (never used but for this) against my scalp […] All visitors, young and old, were similarly measured, and the ritual was to continue for another thirty years.^44^

Evelyn Haythorne discussed her mother’s most treasured possessions: three china cups and saucers decorated in gold and pink, displayed with pride but rarely ever used. Evelyn recalled how on one occasion they were used to signal who was judged suitable for joining the family. Her brother’s new girlfriend admired them but was swiftly told by Evelyn’s disapproving mother that “I only use them for anyone special.”^45^ As well as being functional and decorative, the cups and saucers also served as symbols of family inclusion and exclusion.

For other families in the autobiographies, the risk of losing valuable items through pawning or selling them was a constant threat; the economic realities of life could leave little room for sentimentality. William Woodruff, for example, recalled how in the desperate times of mass unemployment in the interwar period, his family had to sell anything of worth: “even father’s war medals and Jenny’s wedding dress were pawned for food.”^46^ In these cases, an object could go quickly from being a treasured heirloom to being a tradable commodity—and indeed back again, as it was taken to and retrieved from the pawnbrokers in a sometimes endless cycle. An object-based approach, then, allows deeper analysis of the dynamics of family life but also, more specifically, processes of intergenerational transmission and the role of history in everyday life.

## Theme 2: Gender and the Family Archive

Our second theme is the relationship between gender and the family archive. Research in a modern context has identified patterns of gendered behavior that also appeared in the case studies. Pearce has argued that there are distinct gender differences in the way that men and women relate to objects and their significance within a family, suggesting that “it is clear that notions of ‘summing up the family’ and ‘holding memories’ have less emotional significance for men than they do for women.”^47^ Her work also found that prized possessions are passed down the female line in a family, and ultimately, this presents the possibility that “that material culture is matrilineal.”^48^ Similarly, Evans’s study of memory and material culture in colonial Australia found that women were often linked to the handing down of objects associated with the construction and sharing of family trees including diaries and journals; she suggested that “objects have played an important part in the construction of genealogies by women.”^49^

Her study also indicated that while objects of significant economic value might be passed down by men, “more intimate familial objects, of perhaps less obvious economic value, have been deliberately passed down the maternal line of families and usually kept within the home.”^50^ Claire Grey, too, reflecting on her own family’s practice described how the very rituals of sharing history, “gossip” over tea, were by their nature female.^51^ These contemporary gendered practices of family archiving and memory processes have long historical roots, as our case studies show. We also suggest that this strong gendered nature of different archival practices is part of the reason why informal and family archives tend to remain undervalued within the historical discipline.

For the Romans, men appear to have played a major role in the curation and propagation of both items and stories, because of the importance placed on the role these played in preparing elite young men for their future duties in the Roman state.^52^ Women did keep their own archive materials, where their personal and business worlds overlapped; the Babatha archive contains a marriage contract as well as loan paperwork and other legal documentation which gives us an insight into the life of the second-century AD woman that it belonged to. However, while the public display of the *imagines* meant that women inherited family stories as much as their male counterparts, women’s personal archiving appears to have been conceptualized as separate from these larger family narratives.

The deposition of reused Roman items in the graves of early Anglo-Saxon and later medieval adult women and children, but less frequently with adult men, suggests that their use was connected to the construction of a gender identity, or a range of identities, which can be coarsely defined as “nonmasculine.”^53^ It has been suggested that women were largely responsible for the preparation of the deceased for burial in both the Anglo-Saxon and medieval period, and they may therefore have decided which antique family possessions it was appropriate to deposit with the dead and when.^54^ The Anglo-Saxon saucer brooches analyzed by Chris Caple and the repaired or customized Anglo-Saxon brooches studied by Toby Martin show a distinct inclination toward long-term reuse and curation of feminine dress accessories.^55^ At the medieval settlement of Tattenhoe in Buckinghamshire, which was occupied between the late twelfth and late fifteenth centuries, a repaired buckle bore the Lombardic inscription AVEM/ARIA (Ave Maria), which was particularly associated with charms for the protection of women.^56^ The decision to curate and repair this object to extend its life may well have been made by a woman. Silk mesh hairnets from sites in medieval London suggest that highly personal feminine items were passed down within families; thirteenth-century examples in worn condition have been found in mid- to late fourteenth-century contexts.^57^

Some potentially masculine heirlooms have also been discovered archaeologically, although in much smaller numbers. For instance, a brooch from the fifth- to seventh-century cemetery of Loveden Hill in Lincolnshire had been turned into a strap end or mount, possibly for a belt, and may have been altered so that a man could wear it.^58^ Antique Graeco-Egyptian cut gems or Roman cameos, meanwhile, have been found set into rings buried with medieval bishops, such as that of Archbishop Hubert Walter at Canterbury Cathedral.^59^ Meanwhile, at Coppergate in York, a sword pommel of whale bone, dating from the ninth or tenth century, was discovered in a twelfth-century deposit, while an antler tine in a phallic shape, pierced to be worn as an amulet and dated to c.1000 CE, was found in a thirteenth-century deposit.^60^ Both items may have had special significance for male members of a family and been passed down the generations at least in part through the male line.

The twentieth-century autobiographies indicate that both women and men were invested in family archiving practices, deeply treasured particular objects that held emotional or familial significance, and used family memories and histories for particular purposes. Yet there was some evidence that different means of memory-making were of varying significance to men and women. Some women, particularly of older generations, became “keepers” of their families’ stories. For example, Paul Johnson’s mother would repeatedly tell him the story of her life: “It was a kind of verbal autobiography, reaching far back into the past (she was born in 1886) and embracing an enormous cast of characters […] woven skilfully into a continuous and ramifying saga, brought up to data and embroidered with her present discontents.”^61^ For Paul, it was his mother who told him about their family past, legends, and memories that made up their shared family identity. In contrast, Paul’s father was a figure who, in both Paul’s life and as a literary device in the book, communicated information about the history of the place they lived and of communities beyond the family.

Does this reflect the fact that historically women’s lives and work have been more orientated around home and family than men’s? Or perhaps even a different gendered approach to keeping and valuing archives and histories? Jane Hamlett found this true of Victorian middle-class families; in terms of death, for example, while men dealt with the legalities of wills and inheritance, women’s roles in caring for the elderly and laying out the dead often meant they were responsible for the distribution of small, low-value personal items.^62^ The gendered meanings of family archiving practices also differed according to the different kinds of items kept. For example, Jeremy Seabrook described the patterns of memory-making around family photography: “even when the family photographs ceased to be professional and studio-bound, and became a do-it-yourself activity, it was the men who took the pictures, while the women remained custodians of the feelings.”^63^ The practicality of creating family archive items might have been an often masculine practice, while the emotional labor of remembering and passing down stories (and objects associated with them) was frequently that of women. Family archiving is “an art of women.”^64^

In general, female autobiographers in the twentieth-century sample appear to concentrate more on families and relationships, and objects and stories form the focus of their “family archiving.”^65^ Their male counterparts discuss their individual lives, their friends, and their public life in more depth. Male autobiographers were more concerned with preserving their family heritage through passing on the family name or their genealogical research. Andrew Purves, for example, described in detail traditions around naming children after relatives in Scottish borders communities.^66^ Brian Magee wrote of the genealogical research about his family.^67^ Others, such as Edward Short, focused on family names in the wider community.^68^ Yet it was female relatives who frequently told and retold particular stories of family life: Magee’s grandmother, for example, was a key figure in this process.^69^ Like Johnson’s mother and Stafford’s mother, she was the person who communicated particular tales to the next generation of her family.

The stories told by men were also more likely to reflect on how their lives fitted into a wider story of local, regional, and national change. Magee referred to how the Geffrye Museum in London had helped him place his family story in its wider context of the history of working-class life and the city more broadly.^70^ William Woodruff, himself a historian later in life, was the one who wrote up his family history in a formal way as part of a chapter entitled “Family Tree,” but it was his mother who had communicated this to him in his youth. He reflected that “because that kind of thing interested her, she’d made it her business to get it all in her head. Father wasn’t half as good. Family history didn’t interest him—he focused on the present, not the past. Sometimes mother answered my questions about family history in snatches, sometimes at great length. Bit by bit I pieced it together.”^71^ As such, women seem to have played the often crucial role of collecting key objects and telling the stories that went with them, though clearly objects held similar significance for both men and women. Yet it was more likely to be men who formalized this “family history” in writing and placed it in a broader context. The shift from the oral to a written mode of communication reflects the way that the written word has often been privileged over other forms of knowledge formation but records traces of the practices that it replaces and elides.

## Theme 3: Socialization and Identity Formation

A third theme that runs through our case studies follows how family archives were used to transmit a “correct” way to behave and served to inform an individual about how to conduct themselves in terms of their identity as a member of a particular family. There appears to be a consistent intersection between family, identity, and morality, which the family archive plays an important role in mediating. Writing of Victorian England, Hamlett comments that “the long-term significance of domestic things to nineteenth-century families often lay in their role in the transition between generations, as much as in their meaning at the moment of purchase.”^72^ As Hirsch notes of photography, such items can “perpetuate family myths,” thereby creating a sense of cross-generational family identity.^73^ Objects in an archive have the potential to help family members negotiate the intersection between private life, state, and morality.

The preservation of a family archive allows long-dead family members to “speak” to current generations and for people alive now to prepare messages for descendants not yet born. Archives can emphasize particular shared family traits, build a sense of family character or identity, and help family members to learn important moral lessons from each other. The very act of deliberately curating a family history may in and of itself help perpetuate the family in question. Pearce argues that “the creation of material identity is crucial to a family’s sense of wellbeing,” marking out the maintenance of the specifically physical as an important aspect of what keeps families stable across the generations.^74^ Santos and Yan view family history as a means of “living toward mastery of a meaningful life” via the processes of affirmation, negotiation, and maintenance of ongoing identity formation;^75^ the creation of an identity perceived as distinctive, secure, and durable is enabled by the connection to the past.^76^ As such, the passing on of specific objects appears to play as much of a role in the very continuation of a family identity over time as kinship bonds.

The Roman tradition of *exempla* in particular illustrates this theme, since the purpose of handing down stories about ancestors was to preserve examples of admirable behavior. The stories associated with a particular family, along with the wax masks displayed in houses, conveyed strong expectations about the behavior associated with membership of a particular family group. The prominent position of the masks and accompanying wall paintings meant that family members—specifically the boys and adolescents who were their primary intended audience—would walk past them every day and thus have a physical reminder of the lineage that they were supposed to emulate.^77^ In the medieval period, Roberta Gilchrist has noted that items with Christian connotations such as baptismal spoons or *Books of Hours* were treated as heirlooms, while hairnets and hairpins may also have been passed by a mother to her daughter to wear at her wedding.^78^ Arguably, the religious items had a role to play in reminding family members of their duty as Christians, while the hair accessories reminded women of expected wifely behaviors and obligations.

Many families preserve memories because of their relatives’ roles in particular conflicts, thus molding a sense of identity around the bravery shown and sacrifices made by those family members. These associations allow people to tie their small-scale family narratives into national and international history. Alongside commemorating particular moments significant to individual lives, many autobiographers referred to treasured possessions that commemorated or connected them to both world wars. This could be in the form of photographs. Jo Stafford explicitly highlighted how they gave her family a place in a national history: “sepia images in uniform were our link with the Great War; glossier sharper portraits of uncles in uniforms were from the more recent conflict.”^79^ Likewise, William Woodruff’s autobiography starts with a family photograph celebrating his father’s return from the First World War.^80^ Community spaces could also be part of this understanding of the links between family and national histories; numerous war memorials were erected across Britain in tribute to those who had died in the First World War, for example. Edward Short described how, in his village of Warcop, “the controversy about the form our memorial should take was long and bitter.”^81^ The stone monument finally chosen (largely following pressure from the local gentry) symbolized the loss of the community with the now familiar list of names representing each man who had died. Each family placed a wreath or bunch of flowers, a highly emotional moment for the assembled population, and connected their individual loss with a bigger international story.

The inclusion or exclusion of certain figures from a family narrative can be extremely deliberate and part of the process of forming a family’s identity in one direction or another. For the Romans, the creation of an *imago* marked the attainment of a particular political status and was thus a “reward” for political accomplishment;^82^ this would also have meant that a collection of *imagines* would have represented men at roughly the same age, given the age restrictions Roman law placed on holding certain offices. The right to be included in the family history was thus tied inextricably with successful public service. In the twentieth-century autobiographies, Evelyn Cowan told of a local “schnorrer” in her Jewish community in Glasgow, who despite his homeless status, carried with him until his death a whole range of documents—including his “birth certificate, old family snapshots and a letter to be opened in the event of the schnorrer’s death” which proved his family lineage, showing that he was the brother of a notable Glasgow public official.^83^ James Dunn, a missionary worker in the East End of Glasgow, included a photograph of the congratulation letter he received from the church authorities recognizing his long service and contribution in late-nineteenth and early-twentieth century London.^84^ Public service as an identifying familial characteristic thus manifests itself in different social modes. These diverse case studies all record the use of objects and spaces to perpetuate particular family ideals; they could act as a symbolic repository for the intermingling of past, present, and future in ways which will repay further research.

## Theme 4: The Life Course

The final theme that emerges from across the case studies reflects the representation of various markers in the life course of families. The life course differs from chronological or biological aging in that it represents the socially constructed narrative of birth to death that an individual in a society experiences and the various rites of passage and age-related markers that they encounter along the way.^85^ Taking a life-course approach to understanding family archives helps us to understand both the ways that individual cultures respond to culturally constructed moments of the life course and how the transhistorical certainties of birth and death are commemorated and mediated; as Jeremy Seabrook notes of photographs, “[they] induce reflection upon and acceptance of our own mortality, speak of the sameness and continuity of human lives.”^86^ As a practice, family archiving in itself is focused on the preservation of memories of dead relatives and the curation of a particular set of ideals and memories for future relatives. It can display both a pride in the past and a faith in the future.

Viewing objects through a life-course lens helps us appreciate how and when otherwise unremarkable items become important. Objects take on meanings at certain times or because of their association with events and that is as much a part of their value as their function. Items kept in archives thus reflect social attitudes to birth and death across generations but also encapsulate other events viewed as significant rites of passage within a family. These may range broadly from certificates of confirmation to swimming exam certificates, but in their variety, they reflect the priorities of individual families and their valuation of social markers. Objects become embodiments of particular moments of friction, conflict, resolution, and transition that are otherwise lost.

Death is a highly significant theme in all of our research into private archiving practices; objects which are inherited are by definition connected to a dead relative or friend. The transhistorical reality of death can thus become encoded in a particular object. Items are often passed on at the moment of death, either formally through a will or informally through the distribution of personal possessions after someone has died. The things individuals keep can be a crucial way in which they remember someone; as Hamlett has described, “domestic objects could be freighted with new emotional significance in the wake of death.”^87^ It is at this point that the “inalienable” nature of some items crystallizes; mourners may find it easy to let go of some possessions, but others that are intimately associated with the deceased may be much harder, if not impossible, to dispose of in light of their emotional power.

Twentieth-century autobiographers often pointed to the use of objects to connect to a dead loved one such as Molly Hughes’s use of her deceased husband’s furniture and plants. This could be especially important at particularly significant moments in the life course such as Evelyn Cowan’s mother’s wearing of her dead mother’s cameo brooch. In contrast, the deposition of family belongings in Anglo-Saxon graves demonstrates another way in which death might bring new significance to an object, as it passed from circulation among the living to stillness as a grave deposit.

Of course, there are many ways in which families and individuals have found comfort in remembrance of loved ones they have lost. Are objects a different or special way to do so? They provide an important link to loved ones in diverse ways. In the autobiographies studied as part of our third case study, everyday objects that someone had used, such as a table, could help summon them back in spirit; special objects brought out on special occasions could preserve that link at an emotional time. Physical contact with objects that had been in turn touched or used by loved ones seems important. Photographs and letters played a different role; seeing and reading the words of a relative (or friend) could, for some, feel like an even closer way of being near someone who had died, for better or worse. But parallel to this were those autobiographers for whom spaces/places were as evocative a means for remembrance.

The grammar of space is a much-used device in these autobiographies; Pamuk comments that the streets of cities in which we live “will eventually become an index of our emotional landscape.”^88^ Recollection of not only the spaces in which writers had lived, but an account of the writer revisiting those places, is frequently used to open or close an autobiography or make a particular tribute to someone who has died. Ralph Finn, for example, ended his account of his childhood in Aldgate, London, with a description of returning to the streets in which he had grown up. He recalled “seeing” the faces of loved ones who had died: “the street outside was full of bearded old men who looked like my Zaida [grandfather] and the more I stood there the more I felt that the world had swung back on its axis and time had retraced its wild spinning and I was back among the men and women and children I knew.”^89^ Ralph described a similar experience when a friend returned to him an autograph book from his schooldays, filled with quotations and scribblings of his own—these were a “voice from the dead past” and, he added, “there lay my youth.”^90^

Perhaps shared spaces, like homes and streets, could create a more collective history, a shared and public signifier of particular shared past experiences. Spaces can literally transport the person back to feeling like a former self, and this was something both Winifred Foley and Evelyn Cowan described too. Brian Magee, furthermore, recalled his father’s love of going to the theater and music concerts, sitting in his same seat as a monthly subscriber, “which I sometimes, out of pure sentimentality, chose myself during the years immediately after my father’s death in 1947.”^91^ Pure sentimentality, perhaps, but Magee evokes an interesting notion of how inhabiting the same space as a loved one who had died had previously done could create a comforting connection with them.

These episodes all show that it was not just the treasured personal objects that could be used to create a link with the past, such as the special cameo brooch or the daily use of a family table, but macro-objects that were not owned by the family. Magee’s father’s seat in a theater, or more generally the spaces in which relatives had lived, could conjure up the very images of them as living people. Although these spaces are not owned by individuals in the same sense as small objects, considering how the physical occupation of space and physicality on a grand scale speaks to the power of the concrete in a way that goes beyond paperwork. An individual’s class and gender arguably in part influenced this use of objects and spaces. While personal, familial objects seemed to be more treasured by women, men often found other ways to keep alive particular people, memories, and histories including this appropriation of public space and place. Furthermore, working-class writers seem to have viewed particular locations and space as more important for their memories.

One important factor influencing how important personal objects and the preservation of stories and memories were to an individual was their age. Kuhn sees the handing on of traditions as part of a process of creating “collective immortality,” since “a compulsion to tell and retell the same stories, to the point indeed that they become formulaic, has about it the air of a wish to forestall, even evade, death.”^92^ A number of writers reflected on the role personal history played in their older relatives’ lives. Phyllis Willmott described her Gran’s part of their shared family house in London: “pictures, family photographs and china plates covered the walls and an ornately carved and mirrored ‘overmantel’ above the fireplace was crammed on every little shelf with china and brass ornaments—gilt painted jugs, white china swans, coloured glass paper-weights, ugly souvenirs from seaside resorts [etc…] All in all, the front room was a proud exhibition of Gran and Grandad’s pasts, their private museum of treasures.”^93^ In this way, not only did objects create a connection with loved ones who had died, but they also played an important role in creating a particular sense of self rooted in one’s whole life in old age. For an elderly person, keeping a box or a room of treasures which had particular stories and familial (or other) meanings attached to them might be important for a sense of contentment or ease in later life.

While birth and death are the only two moments of human existence that will be universally experienced, family archives also preserve revealing traces of rites of passage. The Roman *imagines*, as we have seen, encapsulate a moment of political success, which was considered vital in the life course of an elite male; there is also evidence suggesting that the practice trickled down to nonelite groups, mainly representing children rather than adult males, thus marking the significance of a different life course stage to a different social group.^94^

Medieval heirlooms can be very closely tied to rites of passage and moments in the life course. The presence of heirloom jewelry, coins, and other objects in children’s graves in the Anglo-Saxon and medieval periods hints at their function in the early stages of life, perhaps marking the achievement of “personhood” for young children. For instance, Anglo-Saxon adult female dress was typically worn by individuals aged over twelve, with some forms of brooch restricted to those over eighteen.^95^ Passing these age thresholds may have been marked by rituals involving the giving and receiving of objects such as jewelry, perhaps including heirloom items, which took on additional significance through being exchanged or gifted at life cycle thresholds. Many of the objects already discussed appear to have been gifted on marriage, such as hairnets and *Books of Hours*, while the apparently masculine heirlooms from Coppergate in York—the phallic amulet and whalebone sword pommel—may have been gifted to mark a young man’s rite of passage relating to a hunt or military training.^96^ Similarly, the curated decorative jugs discovered in a number of medieval households may have been used for drinking rituals on special family occasions that celebrated the life course such as births, betrothals, marriages, and funerals.^97^

Using a life course approach as a way to frame key points in an individual’s life, as identified both by their family and by wider society, allows historians to bring these significant episodes into sharper focus through the objects attached to them. It also offers an opportunity to consider the complexities of these transition points in lived reality. Coming at these issues from the “bottom up,” using objects to look at the specifics of individual life courses and considering how rites of passage, however defined, were marked and remembered, leads to a different perspective on changing family practices over time. The snapshots of lives mediated by individual and familial archive practices offer a different perspective to the traces that may or may not be preserved in official records. This methodology allows us to consider what matters to the individual rather than to organizations or the state.

## Conclusions

The interdisciplinary methodology we have adopted in this article allows us to examine case studies from three individual periods using the full range of research questions found in each other’s specialisms. In this way, the challenges posed by the different conventions of our fields, such as the particular meanings ascribed to the word archive, have become a strength for opening new avenues for enquiry rather than a temptation to seek similarity at the expense of overlooking difference. Interrogating material familiar to us in fresh ways has led us to consider issues such as the way that the intangible family archive manifests in different ways over time and how formally that intangible archive is recognized by our case study cultures. In terms of the objects that have been the focus of this article, juxtaposing our case studies has emphasized the importance of context and situated interpreters and the importance of acknowledging physical items as significant for identity.

Our arguments are threefold. First, we argue that taking families’ material culture, informal archives, and curation practices seriously, we can move further toward a more “democratic” approach to history that values the informal, emotional, and the messy as much as the rational and institutional. Second, this article shows the value of taking a long chronological perspective and interdisciplinary collaboration; talking across conventional academic boundaries has opened up the possibility of reevaluating both memory-making practices and our work as historians. Third, by reframing our activity, we argue that a different understanding of what an archive is enables us to better value those historical forms of knowledge that cannot be found in the institutional archive.

We are aware that in arguing for the importance of viewing material culture in the context of familial archives, we risk rephrasing one of the old fallbacks of archaeology—namely, if you cannot tell what an object’s purpose is or why it is located in its deposit, then the answer must be ritual. We do not suggest that if an item is perplexing, then the reason for that perplexity must lie in some unrecoverable domestic dynamic. That said, our case studies have brought out the importance of considering the familial context in interpreting and understanding artifactual assemblages and understanding the significance of the object for a particular individual.

We hope that the new questions we found ourselves asking of previously familiar material will in turn generate new dialogue within historical periods beyond our case studies. In particular, they offer ways to think about models of interpersonal interaction mediated through physical objects and emphasize the significance of considering multiple points where meanings can be created and renegotiated during an object’s life or within a family archive. Our findings are as relevant to professional archivists as they are to historians, who may find these themes helpful in assisting families to understand their own family archives and the processes that have shaped their formation as part of existing community-focused or community-led curation of content.

This understanding, contemporary or historical, is not clear-cut. The pivot point of all our four themes is that the family archive is not a static or complete “thing” to which objects are added, creating clearly defined chronological strata of deposits for future generations to excavate. Instead, the archive continues to hold and embody the various tensions and stresses between relatives in terms of what enters the archive in the first place, how its presence there is interpreted, and during the process of transferring the archive between keepers. In turn, these tensions reflect the anxieties and priorities of the wider culture within which the family exists.

A broad conception of the family archive can help scholars and archivists reconstruct the significance of objects for social interaction, the preservation of memory, and the formation of identity. The work of the citizen archivist, whether conscious or accidental, plays into larger patterns of shifting conceptions of identity at an individual and familial level. As such, the four themes that this article has uncovered both legitimize the significance of the domestic archive and signal the importance of including objects in the discourse of preservation.

## Notes

1. Orhan Pamuk, *The Innocence of Objects: The Museum of Innocence, Istanbul* (New York: Abrams, 2012), 59.

2. For example, Richard Cox, “Digital Curation and The Citizen Archivist,” in *Proceedings of DigCCurr2009: Digital Curation: Practice, Promises and Prospects*, eds. H. Tibbo, C. Hark, and C. Lee (Chapel Hill, NC: Lulu, 1999), 102–9; Trevor Owens, “Digital Cultural Heritage and the Crowd,” *Curator: The Museum Journal* 56, no. 1 (2013): 121–30; Peter Ride, “Creating #Citizencurators: Putting Twitter into Museum Showcases,” in *Proceedings of the 19th International Symposium of Electronic Art*, eds. K. Cleland, L. Fisher, and R. Harley (Sydney, Australia: University of Sydney, 2013), 1–4. See also public interfaces such as the US National Archives Citizen Archivist Dashboard, last accessed August 26, 2016, https://www.archives.gov/citizen-archivist/.

3. Paul Thompson, *The Voice of the Past* (Oxford, UK: Oxford University Press, 2000), 98–99. For an example of the process of oral family history practices among both Gambian communities and their enslaved relatives and descendants in North America, see Alex Haley, *Roots* (London, UK: Vintage, 1991), 674–9.

4. J. König, K. Oikonomopoulou, and G. D. Woolf, eds., *Ancient Libraries* (Cambridge, UK: Cambridge University Press, 2013).

5. The importance of these shared narratives to Roman identity and political activity cannot be underestimated; G. D. Farney, *Ethnic Identity and Aristocratic Competition in the Roman Republic* (Cambridge, UK: Cambridge University Press, 2007) explores the importance of ethnic identity with the Roman world, while A. M. Gowing, *Empire and Memory: The Representation of the Roman Republic in Imperial Culture* (Cambridge, UK: Cambridge University Press, 2005) discusses the use of memory as a way of constructing the individual self and a wider social identity, particularly when remembering the Republic under the Empire.

6. Katelijn Vandorpe, “Archives and Dossiers,” in *The Oxford Handbook of Papyrology*, eds. R. S. Bagnall (Oxford, UK: Oxford University Press, 2009), 217–8 outlines the various definitions of “archive” used by papyrologists and modern historians; as she notes, the word archive in English is derived from the Greek *archeion*, meaning “government house for official records.”

7. T. Keith Dix, “‘Beware of Promising Your Library to Anyone’: Assembling a Private Library at Rome,” in *Ancient Libraries*, eds. J. König, K. Oikonomopoulou, and G. D. Woolf (Cambridge: Cambridge University Press, 2013, 209-235) traces the growth of Cicero’s private library and the means by which he went about expanding it, including asking other men to borrow volumes from their libraries for his slaves to copy.

8. The frame in which these items are usually discussed is that of cultural memory and its preservation; see, for instance, Catherine Baroin, “Remembering One’s Ancestors, Following in Their Footsteps, Being Like Them: The Role and Forms of Family Memory in the Building of Identity,” in *Children, Memory, and Family Identity in Roman Culture*, eds. Véronique Dasen and Thomas Späth (Oxford, UK: Oxford University Press, 2010, 19-48).

9. A particularly comprehensive study of heirlooms in the medieval period can be found in Chapter 6 of Roberta Gilchrist, *Medieval Life: Archaeology and the Life Course* (Woodbridge: Boydell Press, 2012).

10. Gilchrist, *Medieval Life*, 237–9.

11. Ted Walker, *The High Path* (London, UK: Routledge & Kegan Paul, 1982).

12. Jeremy Seabrook, “My Life Is in that Box,” in *Family Snaps: The Meanings of Domestic Photography*, eds. Patricia Holland and Jo Spence (London, UK: Virago, 1991), 175.

13. Annette Weiner, *Inalienable Possessions: The Paradox of Keeping-While-Giving* (Berkeley: University of California Press, 2005), 6.

14. Vandorpe, “Archives and Dossiers,” 217–9.

15. The archive of Gaius Julius Agrippinus is available electronically through the Leuven Homepage of Papyrus Collections.

16. Allan Jobson, *The Creeping Hours of Time* (London, UK: Robert Hale, 1977), 20–1.

17. The classic work on these artifacts is Harriet I. Flower, *Ancestor Masks and Aristocratic Power in Roman Culture* (Oxford, UK: Clarendon Press, 1996); see also Véronique Dasen, “Remembering One’s Ancestors, Following in Their Footsteps, Being Like Them: The Role and Forms of Family Memory in the Building of Identity,” in *Children, Memory, and Family Identity in Roman Culture*, eds. Véronique Dasen and Thomas Späth (Oxford, UK: Oxford University Press, 2010, 19-48)..

18. See, for instance, Pliny the Elder, *Natural History* 35.7 and Valerius Maximus 5.8.3.

19. David Barr, *Climbing the Ladder: The Struggles and Successes of a Village Lad* (London, UK: Culley, 1910) [available on microfilm at the British Library]; George A Cook, *A Hackney Memory Chest* (London, UK: Centerprise Trust, 1983); Jobson, *The Creeping Hours*, 1977; Edward Short, *I Knew My Place* (London, UK: Macdonald, 1983); Jo Stafford, *Light in the Dust* (Stourbridge, UK: Blake Publishing, 1990/1995).

20. Annette Kuhn, *Family Secrets: Acts of Memory and Imagination* (London, UK: Verso, 2002), 152.

21. Marianne Hirsch, *Family Frames: Photography, Narrative, and Postmemory* (Cambridge, MA: Harvard University Press, 1997), 7–8.

22. Jo Spence, *Putting Myself in the Picture* (London, UK: Camden Press, 1986), 44–5.

23. Stafford, *Light*, 90–1.

24. Ibid., 13.

25. Roger White, *Roman and Celtic Objects From Anglo-Saxon Graves: A Catalogue and Interpretation of Their Use (BAR British Series 191)* (Oxford, UK: British Archaeological Reports, 1988); Helen Eckhardt and Howard Williams, “Objects Without a Past? The Use of Roman Objects in Early Anglo-Saxon Graves,” in *Archaeologies of Remembrance: Death and Memory in Past Societies*, ed. H. Williams (New York: Kluwer Academic, 2003), 141–70; Roberta Gilchrist, “Magic for the Dead? The Archaeology of Magic in Later Medieval Burials,” *Medieval Archaeology* 52 (2008), 119–59.

26. Alison Kyle, “More than Just a Quick Fix? Repair Holes on Early Medieval Souterrain Ware,” in *Make-Do and Mend: Archaeologies of Compromise, Repair and Reuse 9 (BAR International Series 2408)*, eds. B. Jervis and A. Kyle (Oxford, UK: British Archaeological Reports, 2012), 81–96; Toby Martin, “Riveting Biographies: The Theoretical Implications of Early Anglo-Saxon Brooch Repair, Customisation and Use-adaptation,” in *Make-Do and Mend: Archaeologies of Compromise, Repair and Reuse 9 (BAR International Series 2408)*, eds. B. Jervis and A. Kyle (Oxford, UK: British Archaeological Reports, 2012), 53–66; Carole Wheeler, “Beyond a ‘Make-do and Mend’ Mentality: Repair and Reuse of Objects from Two Medieval Village Sites in Buckinghamshire,” in *Make-Do and Mend: Archaeologies of Compromise, Repair and Reuse 9 (BAR International Series 2408)*, eds. B. Jervis and A. Kyle (Oxford, UK: British Archaeological Reports, 2012), 97–106.

27. Gilchrist, *Medieval Life*, 238.

28. Ibid., 249.

29. David Hinton, *Gold and Gilt, Pots and Pins: Possessions and People in Medieval Britain* (Oxford, UK: Oxford University Press, 2005), 331.

30. Chris Caple, “Ancestor Artefacts—Ancestor Materials,” *Oxford Journal of Archaeology* 29, no. 3 (2010), 312–3.

31. Caple, “Ancestor Artefacts,” 314.

32. Katina Lillios, “Objects of Memory: The Ethnography and Archaeology of Heirlooms,” *Journal of Archaeological Method and Theory* 6, no. 3 (1999), 250.

33. Evelyn Cowan, *Spring Remembered: A Scottish Jewish Childhood* (London, UK: Corgi Books, 1990/1974), 92–3; Bryan Magee, *Clouds of Glory: A Hoxton Childhood* (London, UK: Vintage Digital, 2004), 101; Paul Johnson, *The Vanished Landscape: A 1930s Childhood in the Potteries* (London, UK: Phoenix, 2004), 128.

34. Molly Hughes, *A London Family Between the Wars* (Oxford, UK: Oxford University Press, 1940/1979), 48–9.

35. Barr, *Climbing the Ladder*, 38.

36. Bradd Shore, “Making Time for Family: Schemas for Long-term Family Memory,” *Social Indicators Research* 93, no. 1 (2009): 95–103.

37. For examples of the object biography approach, see Arjun Appadurai, “Introduction: Commodities and the Politics of Value,” in *The Social Life of Things: Commodities in Cultural Perspective*, ed. A. Appadurai (Cambridge, UK: Cambridge University Press, 1986), 3–63; Chris Gosden and Yvonne Marshall, “The Cultural Biography of Objects,” *World Archaeology* 31, no. 2 (1999), 169–78; Janet Hoskins, *Biographical Objects: How Things Tell the Stories of People’s Lives* (New York: Routledge, 1998); Lillios, “Objects of Memory,” 1999; Lynn Meskell, *Object Worlds in Ancient Egypt: Material Biographies Past and Present* (Oxford, UK: Berg, 2004).

38. Frank Trentmann, “Materiality in the Future of History: Things, Practices, and Politics,” *Journal of British Studies* 48, no. 2 (2009): 297.

39. For example, see Gilchrist, *Medieval Life*, 243.

40. Pamuk, *The Innocence*, 206.

41. Eckhardt and Williams, “Objects without a Past?” 161.

42. White, *Roman and Celtic Objects*, 161; Eckhardt and Williams, “Objects Without a Past?” 163.

43. Eckhardt and Williams, “Objects without a Past?” 146, 159; Caple, “Ancestor Objects,” 305.

44. Walker, *The High Path*, 10.

45. Evelyn Haythorne, *On Earth to Make the Numbers Up* (Castleford, UK: Yorkshire Art Circus, 1991), 19–20.

46. William Woodruff, *The Road to Nab End: An Extraordinary Northern Childhood* (London, UK: Abacus, 2008/1993), 354–5.

47. Susan Pearce, “The Construction of Heritage: The Domestic Context and its Implications,” *International Journal of Heritage Studies* 4, no. 2 (1998): 93.

48. Pearce, “The Construction of Heritage,” 95.

49. Tanya Evans, “The Use of Memory and Material Culture in the History of the Family in Colonial Australia,” *Journal of Australian Studies* 36, no. 2 (2012): 208.

50. Evans, “The Use of Memory and Material Culture,” 2012.

51. Claire Grey, “Theories of Relativity,” in *Family Snaps: The Meanings of Domestic Photography*, eds. Patricia Holland and Jo Spence (London, UK: Virago, 1991), 107.

52. See Baroin, “Remembering One’s Ancestors,” 2010, for a discussion of how shared family memories, articulated through *exempla* and *imagines*, performed an important civic function.

53. Eckhardt and Williams, “Objects without a Past?” 161; Gilchrist, “Magic for the Dead?” 141–4.

54. Helen Geake “The Control of Burial Practice in Anglo-Saxon England,” in *The Cross Goes North: Processes of Conversion in Northern Europe, AD 300-1300*, eds. M. Carver (Woodbridge, 2003), 264; Gilchrist, “Magic for the Dead?” 151–2; Audrey Meaney, *Anglo-Saxon Amulets and Curing Stones (BAR British Series 96)* (Oxford, UK: Bar Company, 1981), 294–62.

55. Caple, “Ancestor Artefacts,” 2010; Martin, “Riveting Biographies,” 2012.

56. Wheeler, “Beyond a ‘Make-do and Mend’ Mentality,” 98–9; Hinton, *Gold and Gilt*, 226–7.

57. Gilchrist, *Medieval Life*, 240.

58. Martin, “Riveting Biographies,” 59.

59. Gilchrist, “Magic for the Dead?” 141.

60. Gilchrist, *Medieval Life*, 240.

61. Johnson, *The Vanished Landscape*, 3–4.

62. Jane Hamlett, *Material Relations: Domestic Interiors and Middle-class Families in England, 1850-1910* (Manchester, UK: Manchester University Press, 2010), 194.

63. Seabrook, “My Life,” 172.

64. Ibid., 173.

65. Also see Hamlett, *Material Relations*, 17.

66. Andrew Purves, *A Shepherd Remembers* (East Lothian, Scotland: Tuckwell Press, 2001), 8.

67. Magee, *Clouds of Glory*, 87–9.

68. Short, *I Knew My Place*, 10–11.

69. Magee, *Clouds of Glory*, 240.

70. Ibid., 307.

71. Woodruff, *The Road*, 69.

72. Hamlett, *Material Relations*, 12.

73. Hirsch, *Family Frames*, 7.

74. Susan Pearce, “The Construction of Heritage: The Domestic Context and its Implications,” *International Journal of Heritage Studies* 4, no. 2 (1998): 86.

75. Carla A. Santos and Grace Yan, “Genealogical Tourism: A Phenomenological Examination,” *Journal of Travel Research* 49, no. 1 (2010): 56–67.

76. Paul Basu, “My Own Island Home: The Orkney Homecoming,” *Journal of Material Culture* 9, no. 1 (2004): 27–42.

77. See Baroin, “Remembering One’s Ancestors,” 23.

78. Gilchrist, *Medieval Life*, 246, 249.

79. Stafford, *Light*, 91.

80. Woodruff, *The Road*, 1993/2008.

81. Short, *I Knew My Place*, 34–6.

82. Charles Brian Rose, “Forging Identity in the Roman Republic: Trojan Ancestry and Veristic Portraiture,” in *Role Models in the Roman World: Identity and Assimilation*, eds. Sinclair Bell and Inge Lyse Hansen (Ann Arbor: The University of Michigan Press, 2008), 114.

83. Cowan, *Spring Remembered*, 87–8.

84. James Dunn, *From Coal Mine Upwards or, Seventy Years of an Eventful Life* (London, UK: Wildside Press LLC, 1910), 208–9.

85. Mary Harlow and Ray Laurence, *Growing Up and Growing Old in Ancient Rome: A Life Course Approach* (London, UK: Routledge, 2002), 3.

86. Seabrook, “My Life,” 174.

87. Hamlett, *Material Relations*, 180.

88. Pamuk, *The Innocence*, 86.

89. Ralph Finn, *Spring in Aldgate* (London, 1968), 208.

90. Finn, *Spring Remembered*, 183–4.

91. Magee, *Clouds of Glory*, 103.

92. Kuhn, *Family Secrets*, 166.

93. Phyllis Willmott, *Growing Up in a London Village: Family Life between the Wars* (Halifax, UK: Owen, 1979), 26–7.

94. Véronique Dasen, “Wax and Plaster Memories. Children in Elite and Non-elite Strategies,” in *Children, Memory, and Family Identity in Roman Culture*, eds. Véronique Dasen and Thomas Späth (Oxford, 2010), 125.

95. Martin, “Riveting Biographies,” 62.

96. Gilchrist, *Medieval Lives,* 249.

97. Ibid.

